# Supplementary material for: Adsorption of Fluoride onto Acid-Modified Low-Cost Pyrolusite Ore: Adsorption Characteristics and Efficiencies
Source: Int J Environ Res Public Health. 2022 Dec 19;19(24):17103. doi: 10.3390/ijerph192417103 (PMC9779492; doi:10.3390/ijerph192417103)
Supplement: Supplementary file 1 [file ijerph-19-17103-s001.zip › ijerph-2067208-supplementary.pdf]

Supplementary Materials

Adsorption of Fluoride Onto Acid-Modified Low-Cost Pyrolusite Ore: Adsorption Characteristics and Efficiencies

Phacharapol Induvesa [1], Radamanee Rattanakom [2], Sornsiri Sriboonnak [2], Chayakorn Pumas [3,4], Kritsana Duangjan [5], Pharkphum Rakruam [2], Saoharit Nitayavardhana [2], Prattakorn Sittisom [2] and Aunnop Wongrueng [2,3,*]

[1] Bodhivijjalaya College, Srinakharinwirot University, Nakhon Nayok 26120, Thailand
[2] Department of Environmental Engineering, Faculty of Engineering, Chiang Mai University, Chiang Mai 50200, Thailand
[3] Research Center in Bioresources for Agriculture, Industry and Medicine, Chiang Mai University, Chiang Mai 50200, Thailand
[4] Department of Biology, Faculty of Science, Chiang Mai University, Chiang Mai 50200, Thailand
[5] Science and Technology Research Institute, Chiang Mai University, Chiang Mai 50200, Thailand
* Correspondence: aunnop@eng.cmu.ac.th; Tel.: +66-53-94-4101-3; Fax: +66-53-94-4105

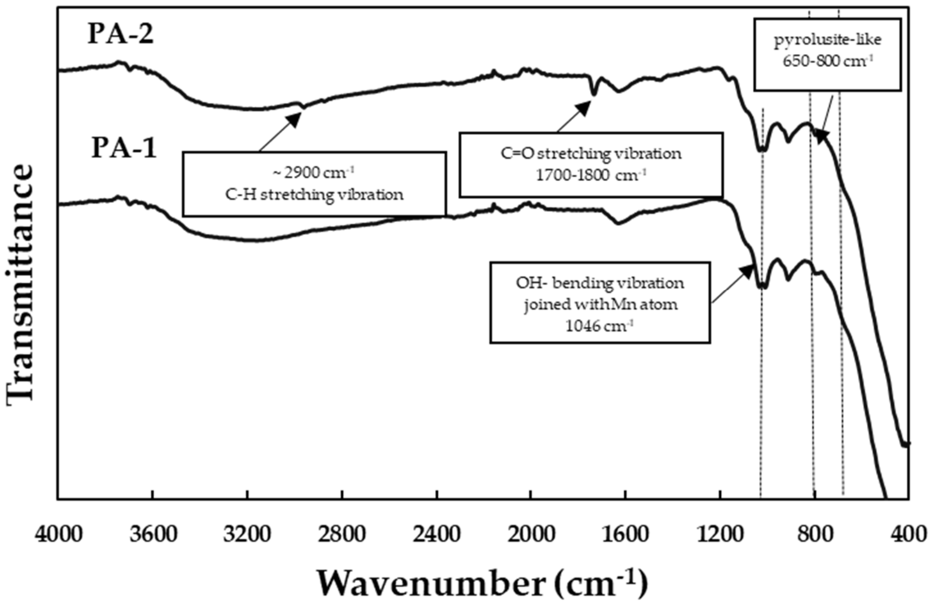

Figure S1. FTIR spectrum of PA-1 and PA-2 (measurement in a range of 400-4000 cm^{-1})

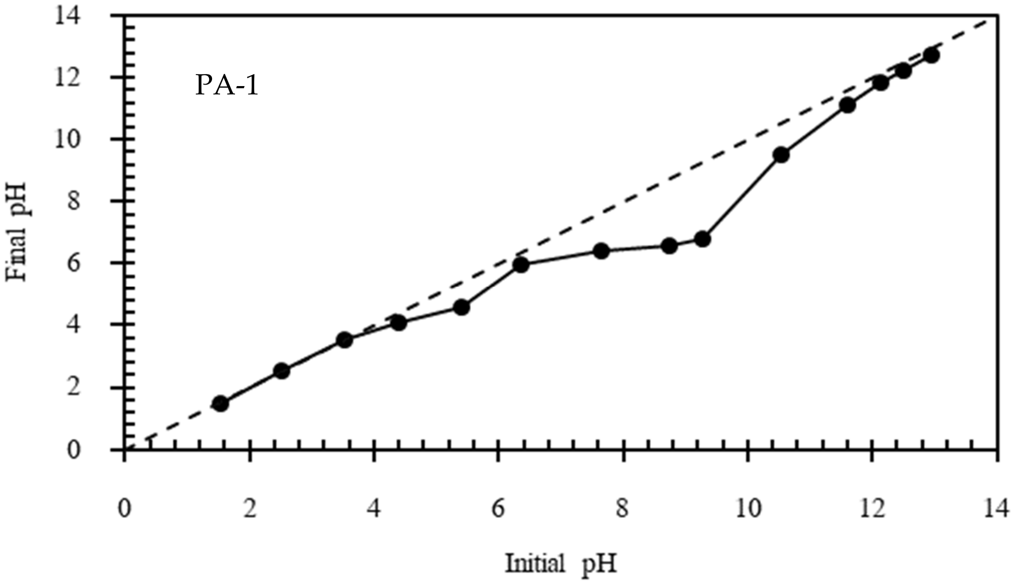

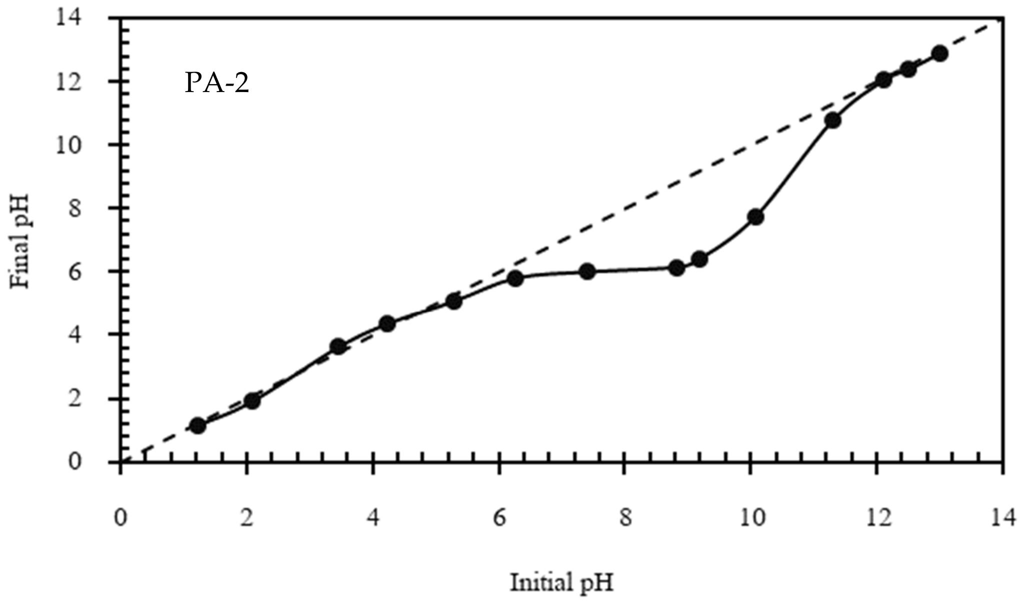

Figure S2. Point of Zero charge of PA-1 and PA-2 (The solid line represents experimental data, and the dashed line represents a pH ratio of 1:1).
